# Aggregation-Induced Emission of Tetraphenylethene-Conjugated Phenanthrene Derivatives and Their Bio-Imaging Applications

**DOI:** 10.3390/nano8090728

**Published:** 2018-09-15

**Authors:** Duy Khuong Mai, Joomin Lee, Ilgi Min, Temmy Pegarro Vales, Kyong-Hoon Choi, Bong Joo Park, Sung Cho, Ho-Joong Kim

**Affiliations:** 1 Department of Chemistry, Chosun University, Gwangju 61452, Korea; maikhuongduy@gmail.com (D.K.M.); valestemmy@gmail.com (T.P.V.); 2 Department of Chemistry, Chonnam National University, Gwangju 61186, Korea; scho@chonnam.ac.kr; 3 Department of Food and Nutrition, Chosun University, Gwangju 61452, Korea; joominlee@chosun.ac.kr; 4 Department of Carbon Materials, Chosun University, Gwangju 61452, Korea; mig@korea.kr; 5 Department of Natural Sciences, Caraga State University, Butuan City 8600, Philippines; 6 Institute of Biomaterial, Kwangwoon University, 20 Kwangwoongil, Nowon-gu, Seoul 01897, Korea; solidchem@hanmail.net (K.-H.C.); bongjoopark@gmail.com (B.J.P.); 7 Department of Electrical & Biological Physics, Kwangwoon University, 20 Kwangwoongil, Nowon-gu, Seoul 01897, Korea

**Keywords:** tetraphenylethene, aggregation, emission, bio-imaging, silica nanoparticles

## Abstract

In this study, a series of rationally designed emissive phenanthrene derivatives were synthesized and their aggregation-induced emission (AIE) properties in tetrahydrofuran (THF)/water mixtures were investigated. Two tetraphenylethene (TPE) segments were conjugated to both ends of the phenanthrene core at the *para*-positions and *meta*-positions, resulting in pTPEP and mTPEP derivatives, respectively. While the TPE-conjugated phenanthrene derivatives did not show any emission when dissolved in pure THF, they showed strong sky-blue emissions in water-THF mixtures, which is attributed to the restriction of intramolecular motions of TPE segments by aggregation. Furthermore, silica nanoparticles loaded with these AIE-active compounds were prepared and proved to be promising intracellular imaging agents.

## 1. Introduction

Several fluorescent nanoparticles with high emission efficiency have been developed in recent years for biological sensing and imaging applications [1]. Typically, emissive molecules have been synthesized using rigid planar aromatic cores because of their good thermal stability, long fluorescence lifetime, and high quantum yield [2,3]. However, conventional fluorescent molecules based on aromatic derivatives suffer from the aggregation caused quenching (ACQ) effect because of π-π stacking interactions in the planar fluorophores, which limits their applicability in organic light-emitting diode (OLED) materials or bio-imaging agents. Recently, Tang and co-workers have pioneered in studying a novel photophysical phenomenon i.e., aggregation-induced emission (AIE), which counteracts the typical effect of the ACQ process [4,5,6,7]. Mechanistic investigations have revealed that the AIE phenomenon is caused by the restriction of intramolecular rotations (IMR) of the luminogens in the aggregate states [8,9,10]. For instance, the four phenyl rings of tetraphenylethene (TPE) derivatives undergo an active IMR in the solution state and are thus non-emissive. In contrast, the IMR process of TPE derivatives is impeded in the aggregate state. Thus, the aggregation states prevent the non-radiative decay channel and elevate these compounds from faint fluorophores to strong blue light emitters [9,11,12]. Following this mechanism, many types of AIE luminogens have been synthesized. In particular, TPE possesses a simple structure but shows a magnificent AIE effect [10,13]. Therefore, TPE and its derivatives have been used to overcome the drawbacks of the ACQ effect and even been applied in biological sciences [8,14,15]. On the other hand, most of TPE derivatives are not soluble in the aqueous media, suggesting that their biological application is limited. However, the fluorescent probes can be made water-soluble and biocompatible by being placed inside nanocarriers such as silica nanoparticles.

Since the first successful synthesis of silica nanoparticles (SNPs) by a scientist working at Mobil [16,17], SNPs have become a promising choice for targeted drug delivery and medical imaging as they are non-toxic and demonstrate good biocompatibility and biodegradability [18,19]. Although SNPs are non-fluorescent, they can act as host materials for bio-imaging agents; fluorescent dyes can be introduced into the silica matrix via physical processes such as sol-gel process.

In this work, we synthesized AIE-active phenanthrene derivatives and investigated their photophysical properties and cell-imaging ability. We conjugated two TPE segments to both ends of the phenanthrene core at the *para*-positions and *meta*-positions to obtain the pTPEP and mTPEP derivatives, respectively. In particular, the phenanthrene has a rather rigid planar structure, thus increase the aggregation ability of their derivatives. The phenanthrene unit can be conjugated with two AIE segments at the *para*-position or *meta*-position, resulting in various AIE-active derivatives. Furthermore, phenanthrene-9,10-diol can be easily alkylated to increase the solubility of AIE-compound and their AIE actives were investigated in tetrahydrofuran (THF)/water mixtures with varying water fraction. The TPEP molecules were also encapsulated in silica nanoparticles. Notably, the entrapped TPEP compounds exhibited AIE behavior with a high emission intensity and the TPEP-loaded silica nanoparticles showed efficient cell-imaging ability. These novel AIE-active compounds and their loading onto silica nanoparticles may provide a useful strategy for the development of promising bio-imaging agents without ACQ.

## 2. Materials and Methods

### 2.1. Materials

Almost of chemicals 9,10-Phenanthrenequinone (PTQ, 99%), trifluoroacetic acid (TFA, 99%), N-iodosuccinimide (95%), iodomethane (CH_3_I, 99%), benzoyl peroxide, bromine (99.99%), nitrobenzene, sodium hydrosulfite (Na_2_S_2_O_4_), dimethyl sulfoxide (DMSO, 99.9%), *n*-butyllithium (2.5 M in hexanes), diphenylmethane (99%), 4-bromobenzophenone (98%), *p*-toluenesulfonic acid monohydrate (98.5%), trimethyl borate (B(OCH_3_)_3_, 99%), tetraethoxysilane (TEOS), (3-aminopropyl) triethoxylsilane (APTES) and ammonium hydroxide (25 wt.% NH_3_ in water) were purchased from Sigma Aldrich (St. Louis, MO, USA). Tetrakis (triphenylphosphine)-palladium(0) (Pd(PPh_3_)_4_, 99%) were purchased from Tokyo chemical industry (Tokyo, Japan). Anhydrous magnesium sulfate (MgSO_4_), potassium hydroxide (KOH), potassium carbonate (K_2_CO_3_) are brought from Daejung chemical (Gyeonggi-do, South Korea). Sodium-metal and benzophenone were used to stir with tetrahydrofuran (THF) for several hours, to remove trace water, then dry THF could be distilled under dry argon before use.

### 2.2. Equipment

Absorption measurements were carried out on a Shimadzu UV-1650 PC spectrometer (Kyoto, Japan) in 1 cm quartz cuvette from 200 to 900 nm. Fluorescence spectra were obtained using a Hitachi F-7000 spectrometer (Tokyo, Japan). Solutions of a concentration of 10 µM were used for both absorption and fluorescence measurements at room temperature (∼25 °C). Absolute quantum yield measurements were carried out using an absolute photoluminescence (PL) quantum yield measurement system (C11347, Hamamatsu Photonics, Hamamatsu, Japan) equipped with a 150-W xenon lamp (Hamamatsu Photonics, Hamamatsu, Japan). All compounds were characterized by ^1^H-NMR spectroscopy on a Bruker AM 250 spectrometer (Billerica, MA, USA). The purity of the products was checked by thin-layer chromatography (TLC, silica gel 60 mesh). The silica nanoparticles were detached by Eppendorf Centrifuge Model 5804. The size and morphology of silica nanoparticles were analyzed by using transmission electron microscopy (TEM) (JEOL, MA, USA). We used machine JEOL-JEM 2100F (Peabody, MA, USA) at an accelerating voltage of 200 kV. To prepare TEM samples, 0.001 wt.% solutions of silica nanoparticles were dropping onto holey carbon film copper. The solvent then was evaporated at room temperature.

### 2.3. Preparation of pTPEP and pTPEP Derivatives

Compound **1** was synthesized according to the reported procedure [20]. Phenanthrenequinone (1.39 g, 6.67 mmol) was placed in 100 mL round bottom flask under nitrogen. 10.0 g of Trifluoromethane-sulfonic acid was added to the flask followed by cooling to 0 °C. N-iodosuccimide (2.99 g, 13.32 mmol) was added slowly over 10 mins. After stirring 6 h at room temperature, the mixture was poured to ice, filtered, and dried to afford a red solid (2.75 g, 89.6% yield). ^1^H NMR (400 MHz, CDCl_3_, δ, ppm): 8.5 (s, 2H), 8.05 (d, 2H), 7.7 (d, 2H).

Compound **4** was synthesized according to the reported procedure [21,22]. A mixture of phenanthrene-9,10-dione (2.00 g, 9.61 mmol), 0.300 g (1.24 mmol) of dibenzoyl peroxide and bromine (3.22 g, 20.15 mmol) were dissolved in nitrobenzene (30 mL). The mixture was irradiated with Tungsten lamp for 1 h, then heated to 120 °C for 2 h. The reaction was then cooled to room temperature and 30 mL of hexane was added. The precipitated product was filtered and washed with hexane. After drying of the precipitates, 3,6-dibromo phenanthrene-9,10-dione was obtained as an orange powder (2.63 g, 74.8% yield). ^1^H NMR (400 MHz, CDCl_3_, δ, ppm): δ 8.13 (d, 4H), 7.69 (d, 2H).

Compounds **2** and **5** were synthesized using the same procedure. A representative example is described for compound **2**. 2.75 g (5.98 mmol) of compound **1** and 3.67 g (21.08 mmol) of Na_2_S_2_O_4_ were added in a mixture of THF/H_2_O (150/50 mL). The mixture was stirred at room temperature for 6 h then extracted with THF and H_2_O. The organic layer was filtered, dried over anhydrous magnesium sulfate then evaporated to get **2** (2.68 g, 97.1% yield). ^1^H NMR (400 MHz, DMSO, δ, ppm): δ 9.28 (s, 2H, ArOH), 8.50 (s, 2H, Ar-H), 8.48 (d, 2H, Ar-H), 7.80 (d, 2H, Ar-H).

Compounds **3** and **6** were synthesized using the same procedure. A representative example is described for compound **3**. Compounds **2** (5.00 g, 10.82 mmol) was dissolved in DMSO (50 mL). After addition of 2.43 g (43.31 mmol) of pulverized potassium hydroxide, 6.14 g of methyl iodide was added dropwise under ice cooling over 20 mins. The mixture was stirred for 12 h at room temperature [23], then the resulting mixture was poured into 100 mL of water and extracted with diethyl ether. The organic phase was dried over anhydrous magnesium sulfate and filtered. The solvent was removed in a rotary evaporator. The crude product **3** was purified by column chromatography (dichloromethane: *n*-hexane by the ratio 2:1, *v*/*v*) to get yellow solid (3.03 g, 57.1% yield). ^1^H NMR (400 MHz, CDCl_3_, δ, ppm): δ 8.58 (s, 2H), 8.26 (d, 2H), 7.86 (d, 2H), 4.07 (s, 6H). Compound **3** was obtained as the orange solid (497 mg, 59.7% yield). ^1^H NMR (400 MHz, CDCl_3_, δ, ppm): δ 8.65 (s, 2H), 8.08 (d, 2H), 7.74 (d, 2H).

### 2.4. General Procedure for the Suzuki Coupling for the Preparation of pTPEP and mTPEP

TPE-boronic acid was synthesized according to the reported procedure [10]. In a 100 mL round bottom flask was placed on a mixture of compound 3 (500 mg, 1.02 mmol), Pd(PPh_3_)_4_ (50 mg, 0.043 mmol), TPE-B(OH)_2_ (845 mg, 2.24 mmol) and K_2_CO_3_ (1.38 g) with the mixture of toluene/ethanol/water by the ratio 40/5/5 mL under argon air. The reaction was refluxed for 12 h then being cooled to room temperature. The reactant mixture was washed three times by water and dichloromethane, dried over anhydrous magnesium sulfate and filtered then was concentrated by rotary evaporation. The pTPEP was purified by column chromatography, in which hexane/dichloromethane (3:1, *v*/*v*) was used as the eluent to obtain a white solid (505 mg, 55.1% yield). ^1^H NMR (400 MHz, CDCl_3_, δ, ppm): δ 8.62 (d, 2H), 8.4 (s, 2H), 7.81 (d, 2H), 7.55 (d, 4H), 7.05–7.18 (m, 34H), 4.11 (s, 6H).

The mTPEP was synthesized according to the same procedure for compound pTPEP. The final compound was collected as the white solid (586 mg, 52.2% yield). ^1^H NMR (400 MHz, CDCl_3_, δ, ppm): δ 8.82 (s, 2H), 8.24 (d, 2H), 7.82 (d, 2H), 7.54 (d, 4H), 7.05–7.19 (m, 34H), 4.06 (s, 6H).

### 2.5. Synthesis of TPEP Derivatives-Loaded Silica Nanoparticles (mTPEP-SiO_2_ and pTPEP-SiO_2_)

Briefly, TPEP derivatives were loaded into silica nanoparticles (TPEP-SiO_2_) according to the modified synthetic procedures [24]. In 100 mL round bottom flask, a mixture of 1 mL of TEOS, 32 mL of ethanol and 0.26 mL of TPEP derivatives solution in ethanol were added. The mixture was stirred for 1 h then the aqueous solution of NH_4_OH (25%, 1.5 mL) was added to the mixture and stirred for 36 h. Finally, the resulting mixtures were centrifuged at 10,000 rpm for 10 min and washed three times with distilled water.

### 2.6. Entrapment Efficiency

The entrapment efficiency was determined based on the calculation of amount of un-reacted TPEP derivatives in the supernatant. The concentration of free TPEP derivatives was calculated by UV spectrometer. Following the previously reported method [25], the entrapment efficiency (E %) was confirmed by Equation (1) in which the total concentration of TPEP derivatives [mTPEP]_0_, [pTPEP]_0_ and that concentration was in the supernatant [mTPEP]_s_, [pTPEP]_s_.

E_m_ % = {([mTPEP]_0_ − [mTPEP]_s_)/[mTPEP]_0_} × 100 (1)

### 2.7. Photostability of Nanoparticles

To study the photostability of free TPEP derivatives, a 3.5 mL of EtOH solution contains 1 mg of TPEP derivatives which was putted in a quartz cuvette in the dark at the room temperature. The TPEP-loaded silica nanoparticles were also prepared with the similar concentration condition in EtOH solution. Photobleaching experiments were carried out by irradiating the samples with Xe lamp (150 W, Abet Technologies, Milford, CT, USA). At every 10 mins of irradiation, the absorption spectra of each sample were measured by UV-Vis spectrophotometer.

### 2.8. Cell Culture and Bio-Imaging Experiment

To assess the cellular uptake and imaging abilities of mTPEP-SiO_2_ and pTPEP-SiO_2_ nanoparticles, HeLa (human cervix adenocarcinoma) was obtained from the American Type Culture and Collection and cultured in Dulbecco’s modified Eagle’s medium (DMEM, Welgene, Gyeongsangbuk-do, South Korea) with 10% fetal bovine serum (FBS, Welgene, Gyeongsangbuk-do, South Korea) and 1% penicillin-streptomycin under a humidified atmosphere (containing 5% CO_2_ and 95% air) at 37 °C. Pre-cultured HeLa cell was plated in a 24-well cell culture plate at 1 × 10^5^ cells/mL and incubated at 37 °C in 5% CO_2_ for 24 h [26]. After incubation, the media of each well in the plate were changed with fresh media and further incubated with 40 μg/mL of mTPEP-SiO_2_ and pTPEP-SiO_2_ nanoparticles for 2 h in the dark condition. Finally, the plate was washed three times with Dulbecco’s phosphate buffered saline (DPBS, Welgene, Gyeongsangbuk-do, South Korea) to remove remained silica nanoparticles before cellular imaging [27]. The morphology of HeLa cells was observed under an automated live cell imager (Lionheart FX, BioTek Instruments, Inc., Winooski, VT, USA) with 20× objective lens and fluorescence optics (excitation at 377 nm and emission at 447 nm).

## 3. Results and Discussion

### 3.1. Synthesis and Optical Properties

**Scheme 1:**
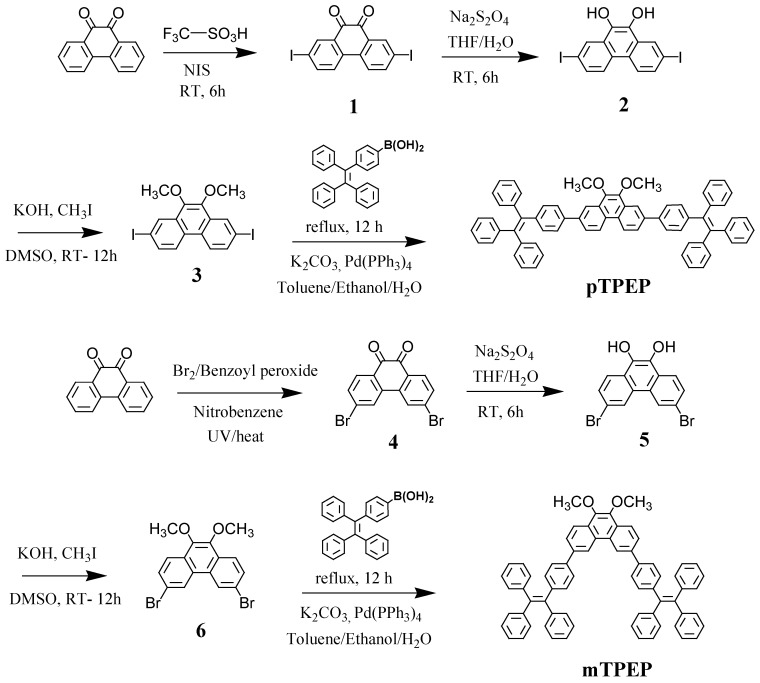
Synthesis of pTPEP and mTPEP.

Scheme 1 outlines the synthesis of TPEP derivatives consisting of a phenanthrene moiety and two TPE units. The two TPE segments were conjugated to both ends of the phenanthrene core in the *para*-positions and *meta*-positions to obtain pTPEP and mTPEP, respectively. The synthesis of the aromatic core started with the halogenation of phenanthrenequinone to yield intermediates 2,7-diiodophenanthrenequinone (**1**) and 3,6-dibromophenanthrenequinone (**4**), which were then reduced to 2,7-diiodophenanthrene-9,10-diol (**2**) and 3,6-dibromophenanthrene-9,10-diol (**5**), respectively, using sodium hydrosulfite. The etherification of **2** and **5** with iodomethane in the presence of potassium hydroxide was performed and subsequent Sonogashira reactions with TPE-boronic acid produced the pTPEP and mTPEP derivatives, respectively.

The photophysical properties of the TPEP derivatives were investigated in various solvents. The mTPEP compound shows absorption peaks (λ_ab_) in the range of 329–336 nm, which are slightly red-shifted with an increase in the solvent polarity but not significant (Table 1). On the other hand, the pTPEP compound exhibits two main absorption peaks. The higher-energetic peaks observed in the 308–312 nm region could be assigned to the local transition of the phenanthrene core while the lower-energetic peaks in the region 345–355 nm were attributed to the fully delocalized π-π* transition between the highest occupied molecular orbital (HOMO) and lowest unoccupied molecular orbital (LUMO) of pTPEP [2]. The overall red-shifted absorption of pTPEP as compared to that of mTPEP is likely responsible for the 1-dimensionally extended π-conjugation of the *para*-linked TPE segments mediated through phenanthrene core relative to their counterpart with the *meta*-linked TPEs [28].

**Figure 1:**
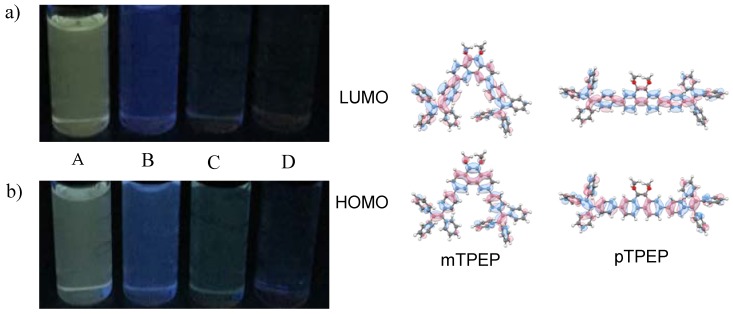
Photographs of (**a**) mTPEP and (**b**) pTPEP under UV illumination in different solvents: A hexane, B dichloromethane, C THF, and D ethanol; and the frontier molecular orbitals.

**Figure 2:**
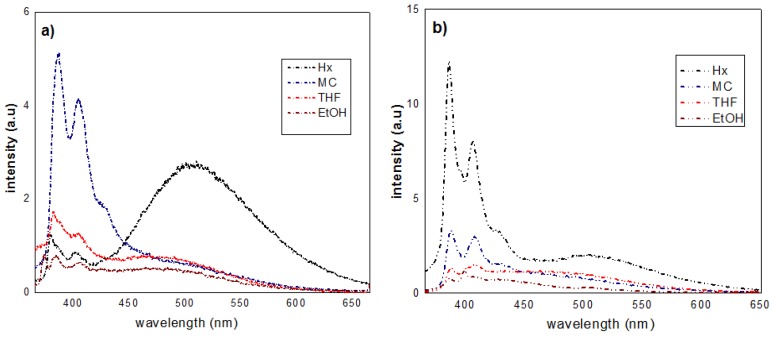
Emission spectra of solutions of (**a**) mTPEP and (**b**) pTPEP derivatives in solvent with different of polarities. Solution concentration: 10 µM. Excitation at their absorption maximum.

The luminescence behaviors of the TPEP derivatives varied dramatically on varying the solvents in contrast to their UV-Vis spectra. Upon excitation by UV light at 365 nm, the TPEP derivatives in non-polar hexane displayed yellow-green emissions (Figure 1), which were deep blue in relatively polar solvents such as dichloromethane and nearly invisible in polar media such as THF and ethanol. In their emission spectra, the TPEP derivatives showed multi-peaks at 380–410 nm, which reduced in intensity as the solvent polarity increased (Figure 2). The TPE units are well-known for poor fluorescence in their dissolved states because of the loss of excitation energy by IMR of the four phenyl rings [2]. Similarly, in this study, the multiple phenyl rings in the isolated molecules of mTPEP and pTPEP luminogens underwent active IMR, which effectively annihilated their excited states and rendered them non-emissive [7,8,29,30,31]. Notably, a strong peak appeared at ~480 nm in the non-polar hexane solution corresponding to the emission from aggregated TPE units. The low solubility of the TPEP derivatives in hexane seems to cause the aggregation of TPE units. The restrain on IMR of TPE units due to their aggregation enhanced the emission intensity at ~480 nm, suggesting that the TPEP compounds exhibited AIE behavior in hexane solution.

### 3.2. AIE Properties

**Figure 3:**
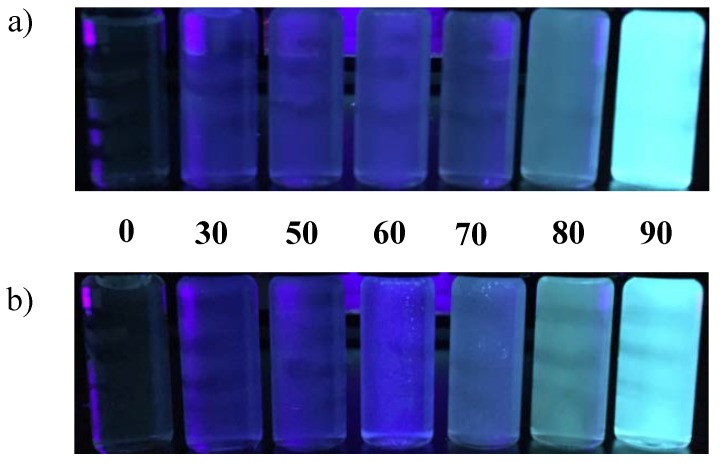
Photographs of (**a**) mTPEP and (**b**) pTPEP in THF/water mixture with different fraction of water taken under UV light (365 nm).

To further confirm the AIE properties of the TPEP derivatives, we investigated their optical properties in THF/water mixtures having different water fractions. Since TPEP derivatives are not soluble in water, they aggregated in the THF/water solutions with high water fractions. As shown in the photographs of the THF/water solutions of the TPEP derivatives taken under UV illumination (365 nm) (Figure 3), the colors became deep blue when the water fraction increased to 60% and turned strong sky blue when the volume of water was 90%.

**Figure 4:**
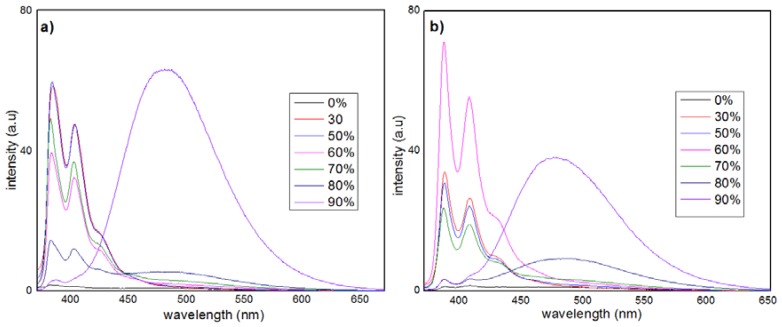
Emission spectra of (**a**) mTPEP and (**b**) pTPEP in THF/water mixtures at excitation wavelengths of 355 nm and 333 nm, respectively. The concentration of solution is 10 µM.

The emission profiles of the TPEP compounds were consistent with their corresponding photographs. Almost no emission signal could be detected from the TPEP compounds in pure THF. When the water fraction was 60%, the emission peaks at ~380–410 nm became stronger (Figure 4). This was attributed to the enhanced hydrophobicity of the media because of the aggregation of the TPEP compounds in the poor solvent (water). Strong emissions at ~480 nm were observed when the water fraction reached 70%, which intensified ~51 and 27-fold, respectively for mTPEP and pTPEP when the water fraction was 90% as compared to the emissions from pure THF solutions. These results confirmed that the TPE segments started to aggregate in solutions comprising over 70% of water, resulting in the restriction of the IMR of TPE units that caused AIE.

### 3.3. Synthesis and Characterization of TPEP-SiO_2_ Nanoparticles

#### 3.3.1. Size Determination

**Figure 5:**
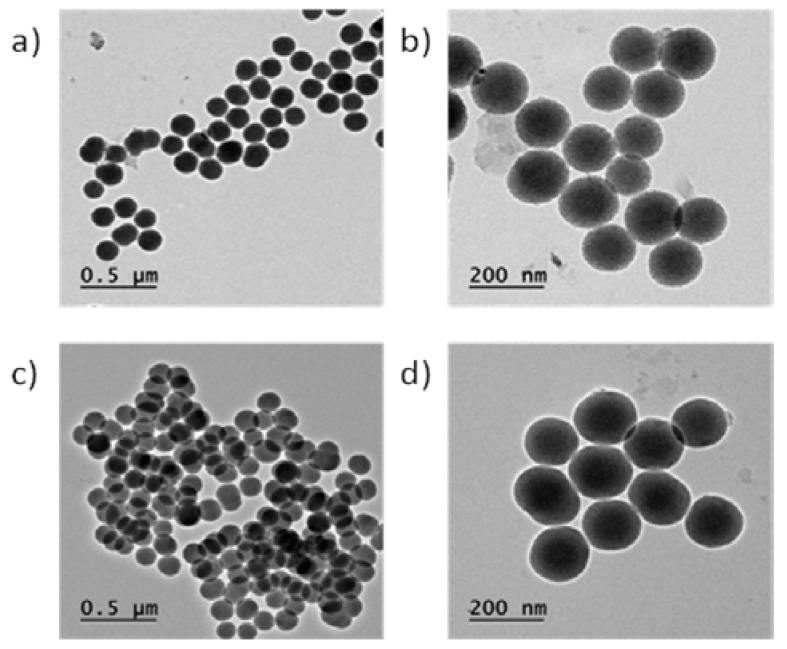
TEM images of silica nanoparticles, (**a**,**b**) mTPEP-SiO_2_ and (**c**,**d**) pTPEP-SiO_2_.

**Figure 6:**
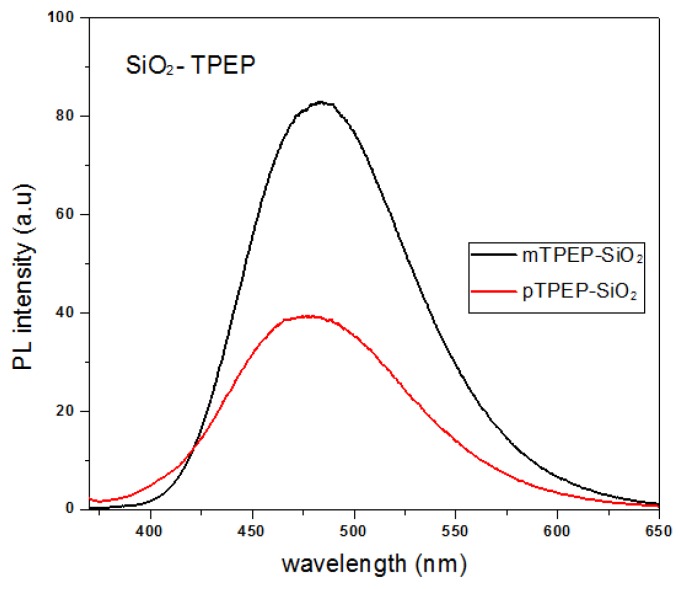
Emission spectra of mTPEP-SiO_2_ and pTPEP-SiO_2_ in water at excitation wavelengths of 355 and 333 nm, respectively. The concentration of silica nanoparticles in water is 2.0 mg/mL.

We also prepared silica nanoparticles containing TPEP compounds via a one-pot synthesis method to investigate their AIE behaviors within silica matrix in water. The TPEP-loaded silica nanoparticles, TPEP-SiO_2_, were prepared by hydrolysis and condensation of TEOS in the presence of alcoholic TPEP solution. The TEM images showed that the synthesized SiO_2_ nanoparticles were spherical with a narrow size distribution from 110 to 130 nm (Figure 5). Additionally, it was found that the size and morphology of both mTPEP-loaded and pTPEP-loaded nanoparticles were very similar. The entrapment efficiencies of the TPEP compounds in the SiO_2_ nanoparticles were calculated using Equation (1). The entrapment yields of TPEP derivatives were calculated to be 99.1% and 99.8% for mTPEP-SiO_2_ and pTPEP-SiO_2_, respectively. Also, the loading efficiencies of the TPE derivatives into the nanoparticles were ~0.77% *w*/*w* and 0.87% *w*/*w* of the total weight of the nanoparticles for mTPEP-SiO_2_ and pTPEP-SiO_2_, respectively. Importantly, mTPEP-SiO_2_ and mTPEP-SiO_2_ in aqueous solutions exhibited strong emissions at 477 nm and 482 nm, respectively, as shown in Figure 6, which was the same as the results observed for TPEP derivatives in THF/water mixture. Fluorescence quantum yields of mTPEP-SiO_2_ and pTPEP-SiO_2_ increased up to 0.191 and 0.176, respectively. This result suggests that the TPEP derivatives aggregated inside the silica matrix and formed the AIE-active silica nanoparticles in the aqueous solution.

#### 3.3.2. Photobleaching

**Figure 7:**
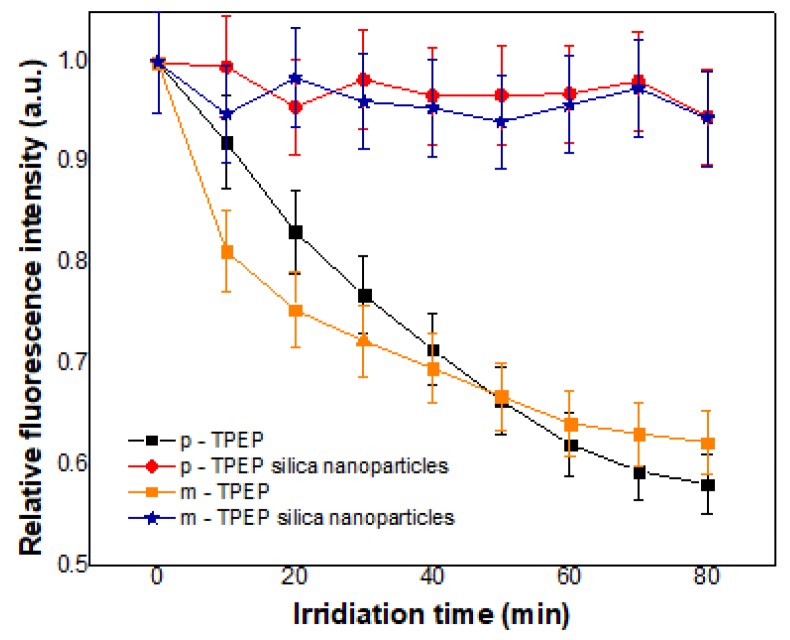
Irradiation time dependent photobleaching behaviors of silica nanoparticles versus free dye of pTPEP and mTPEP under Xe lamp (150 W).

The photostability of silica nanoparticles is also important as they are to be used as bio-imaging agents in complex physiological environments [32]. In this respect, to verify the photostability of the prepared emissive silica nanoparticles, the photo-degradation experiments of TPEP-SiO_2_ nanoparticles and pure compounds were carried out under Xe lamp irradiation (150 W) three times. Figure 7 shows the photobleaching behavior of the TPEP-SiO_2_ nanoparticles and free TPEP derivatives. After 80 min of irradiation of the Xe lamp, the fluorescence intensity of pTPEP and mTPEP compounds decreased to 58% and 64%, respectively, compared to the initial intensity. On the other hand, the TPEP-SiO_2_ nanoparticles did not show any obvious changes in fluorescence intensity even after continuous Xe lamp irradiation for 80 min. From the observed results, it was evident that the TPEP-SiO_2_ nanoparticles had high photostability. The silica matrix can protect the encapsulated TPEP molecules by making them inaccessible to the solvent and reduce their oxidation by lowering the diffusion rate of oxygen, which results in the enhanced photostability. Furthermore, the aggregation of TPEP molecules could prevent further photobleaching and photo-oxidation by preventing oxygen diffusion into the nanoaggregates within the silica matrix [27]. These results indicate that the TPEP derivatives encapsulated in the silica matrix have enhanced the potential for bio-imaging applications because a high photostability of fluorescent probes benefits their long-term cell imaging.

### 3.4. TPEP-SiO_2_ for Cell Imaging

**Figure 8:**
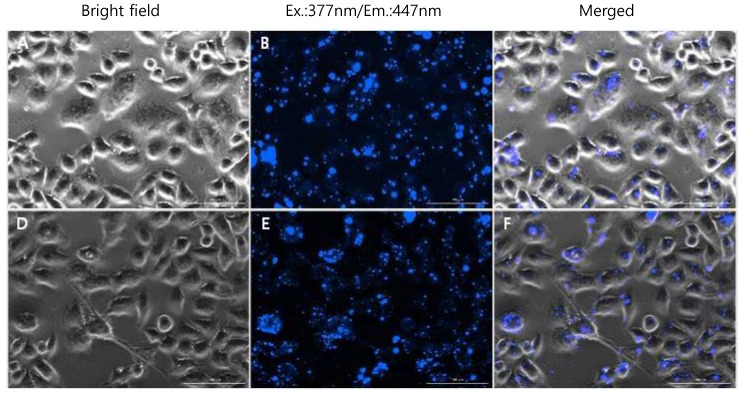
Cellular images of HeLa cells treated with the pTPEP-SiO_2_ (**A**–**C**) and mTPEP-SiO_2_ (**D**–**F**) nanoparticles. The images of HeLa cells were taken with a 20× optical lens and fluorescence optics (excitation at 377 nm and emission at 447 nm) after 2 h incubation with 40 μg/mL of mTPEP-NPs and pTPEP-NPs in the dark condition. Scale bar represents 100 μm.

To investigate the effectiveness of the fluorescent nanoparticles for cellular imaging, HeLa cells were treated with the TPEP-encapsulated nanoparticles. Sky blue-colored emission was observed when the loaded nanoparticles were excited at 377 nm as shown in Figure 8B,E. The images in Figure 8B,E show the cellular uptake and intracellular distribution of mTPEP-SiO_2_ and pTPEP-SiO_2_ and exhibit the successful cellular imaging of the HeLa cells without any morphological change and cytotoxicity for 2 h. This result is consistent with the reported nano-silica characteristics. Based on this result, it was evident that the mTPEP and pTPEP encapsulated inside silica nanoparticles would be useful as materials for cellular imaging.

## 4. Conclusions

In conclusion, TPE-modified phenanthrene derivatives were synthesized and their solvent-dependent AIE properties were investigated. Two TPE units were conjugated to both ends of the phenanthrene core at the *meta*-positions and *para*-positions to obtain mTPEP and pTPEP derivatives, respectively. Investigation of the photophysical properties revealed that the TPEP derivatives exhibited AIE properties in the THF/water mixtures; the derivatives emitted strong sky-blue light until the fraction of water reached up to 90%. This was attributed to the suppressed rotation of the four phenyl rings of the TPE units, which prevented the non-radiative decay process and increased the emission intensity. Furthermore, the two TPEP compounds were encapsulated into silica nanoparticles; they adopted a spherical shape and were mono-disperse with diameters ranging ~110 nm. The TPEP-loaded silica nanoparticles emitted strong sky-blue emission in aqueous solutions and fluorescently visualized HeLa cells because AIE-activity of the TPEP compounds was also achieved within the silica matrix. Notably, the silica matrix acts as a protective shield, enhancing the photostability of the entrapped AIE compounds. Thus, these novel AIE-active compounds and their loading onto silica nanoparticles may provide a useful strategy for the development of promising bio-imaging agents without ACQ behavior.

## Figures and Tables

**Table 1:** Optical transition of two TPEP derivatives in different solvents.

**Solvent**	mTPEP			pTPEP		
	λ_ab_ (nm)	λ_em_ (nm)	*Φ*_F_	λ_ab_ (nm)	λ_em_ (nm)	*Φ*_F_
Hexane	329	377/401	0.016	308/345	384/406	0.043
THF	333	386/403	0.005	312/355	386/408	0.005
Dichloromethane	331	385/402	0.005	310/346	389/407	0.006
Ethanol	336	380/404	0.005	nd	382/398	nd

λ_ab_, absorption maximum; λ_em_, emission maximum; *Φ*_F_, absolute fluorescence quantum yield; nd, no determination. Emission spectra were measured by irradiating the solution (10 µM) at their absorption maximum.

## References

[1] Lee S.H., Hoa T.H., Temmy P.V., Cho S., Kim H.J. (2016). Multi-color fluorescence of pNIPAM-Based nanogels modulated by dual stimuli-responsive FRET processes. Dyes Pigments.

[2] Jadhav T., Choi J.M., Lee J.Y., Dhokale B., Misra R. (2016). Non-doped blue organic light emitting devices based on tetraphenylethylene-π-imidazole derivatives. Org. Electron..

[3] Tang X., Bai Q., Peng Q., Gao Y., Li J., Liu Y., Yao L., Lu P., Yang B., Ma Y. (2015). Efficient deep blue electroluminescence with an external quantum efficiency of 6.8% and CIE_y_ < 0.08 based on a phenanthroimidazole-sulfone hybrid donor-acceptor molecule. Chem. Mater..

[4] Grimsdale A.C., Leok Chan K., Martin R.E., Jokisz P.G., Holmes A.B. (2009). Synthesis of light-emitting conjugated polymers for applications in electroluminescent devices. Chem. Rev..

[5] Zong L., Xie Y., Wang C., Li J.-R., Li Q., Li Z. (2016). From ACQ to AIE: The suppression of the strong π–π interaction of naphthalene diimide derivatives through the adjustment of their flexible chains. Chem. Commun..

[6] Vendrell M., Zhai D., Er J.C., Chang Y.-T. (2012). Combinatorial strategies in fluorescent probe development. Chem. Rev..

[7] Luo J., Xie Z., Lam J.W.Y., Cheng L., Chen H., Qiu C., Kwok H.S., Zhan X., Liu Y., Zhu D. (2001). Aggregation-induced emission of 1-methyl-1,2,3,4,5-pentaphenylsilole. Chem. Commun..

[8] Faisal M., Hong Y., Liu J., Yu Y., Lam J.W.Y., Qin A., Lu P., Tang B.Z. (2010). Fabrication of fluorescent silica nanoparticles hybridized with AIE luminogens and exploration of their applications as nanobiosensors in intracellular imaging. Chem. Eur. J..

[9] Zhao Z., Chen S., Shen X., Mahtab F., Yu Y., Lu P., Lam J.W.Y., Kwok H.S., Tang B.Z. (2010). Aggregation-induced emission, self-assembly and electroluminescence of 4,4’-bis(1,2,2-triphenylvinyl)biphenyl. Chem. Commun..

[10] Zhao Z., Chen S., Chan C.Y.K., Lam J.W.Y., Jim C.K.W., Liu P., Chang Z., Kwok H.S., Qiu H., Tang B.Z. (2012). A facile and versatile approach to efficient luminescent materials for applications in organic light-emitting diodes. Chem. Asian J..

[11] Yu G., Yin S., Liu Y., Chen J., Xu X., Sun X., Ma D., Zhan X., Peng Q., Shuai Z. (2005). Structures, electronic states, photoluminescence, and carrier transport properties of 1,1-disubstituted 2,3,4,5-tetraphenylsiloles. J. Am. Chem. Soc..

[12] Dong Y., Lam J.W.Y., Qin A., Liu J., Li Z., Tang B.Z., Sun J., Kwok H.S. (2007). Aggregation-induced emissions of tetraphenylethene derivatives and their utilities as chemical vapor sensors and in organic light-emitting diodes. Appl. Phys. Lett..

[13] Yang J., Huang J., Li Q., Li Z. (2016). Blue AIEgens: approaches to control the intramolecular conjugation and the optimized performance of OLED devices. J. Mater. Chem. C.

[14] Wang M., Zhang G., Zhang D., Zhu D., Tang B.Z. (2010). Fluorescent bio/chemosensors based on silole and tetraphenylethene luminogens with aggregation-induced emission feature. J. Mater. Chem..

[15] Liu Y., Yu Y., Lam J.W.Y., Hong Y., Faisal M., Yuan W.Z., Tang B.Z. (2010). Simple biosensor with high selectivity and sensitivity: Thiol-specific biomolecular probing and intracellular imaging by AIE fluorogen on a TLC plate through a Thiol-Ene click mechanism. Chem. Eur. J..

[16] Beck J.S., Vartuli J.C., Roth W.J., Leonowicz M.E., Kresge C.T., Schmitt K.D., Chu C.T.W., Olson D.H., Sheppard E.W., McCullen S.B. (1992). A new family of mesoporous molecular sieves prepared with liquid crystal templates. J. Am. Chem. Soc..

[17] Wang Z., Hong X., Zong S., Tang C., Cui Y., Zheng Q. (2015). Bodipy-doped silica nanoparticles with reduced dye leakage and enhanced singlet oxygen generation. Sci. Rep..

[18] Kim J., Lee J.E., Lee J., Jang Y., Kim S.-W., An K., Yu J.H., Hyeon T. (2006). Generalized fabrication of multifunctional nanoparticle assemblies on silica spheres. Angew. Chem..

[19] Lee J.E., Lee N., Kim H., Kim J., Choi S.H., Kim J.H., Kim T., Song I.C., Park S.P., Moon W.K. (2010). Uniform mesoporous dye-doped silica nanoparticles decorated with multiple magnetite nanocrystals for simultaneous enhanced magnetic resonance imaging, fluorescence imaging, and drug delivery. J. Am. Chem. Soc..

[20] Kim H.-J., Jung E.-Y., Jin L.Y., Lee M. (2008). Solution behavior of dendrimer-coated rodlike coordination polymers. Macromolecules.

[21] Kobin B., Grubert L., Blumstengel S., Henneberger F., Hecht S. (2012). Vacuum-processable ladder-type oligophenylenes for organic-inorganic hybrid structures: Synthesis, optical and electrochemical properties upon increasing planarization as well as thin film growth. J. Mater. Chem..

[22] Brunner K., van Dijken A., Börner H., Bastiaansen J.J.A.M., Kiggen N.M.M., Langeveld B.M.W. (2004). Carbazole compounds as host materials for triplet emitters in organic light-emitting diodes:  Tuning the homo level without influencing the triplet energy in small molecules. J. Am. Chem. Soc..

[23] Speck M., Niethammer D., Senge M.O. (2002). Isomeric porphyrin phenanthrenequinones: Synthesis, NMR spectroscopy, electrochemical properties, and in situ EPR/ENDOR studies of the o-semiquinone anion radicals. J. Chem. Soc. Perk. T. 2.

[24] De Oliveira L.F., Bouchmella K., Picco A.S., Capeletti L.B., Gonçalves K.A., Santos J.H.Z.D., Kobarg J., Cardoso M.B. (2017). Tailored silica nanoparticles surface to increase drug load and enhance bactericidal response. J. Braz. Chem. Soc..

[25] Kardys A.Y., Bharali D.J., Mousa S.A. (2013). Amino-functionalized silica nanoparticles: In vitro evaluation for targeted delivery and therapy of pancreatic cancer. J. Nanotechnol..

[26] Wu G., Zeng F., Wu S. (2013). A water-soluble and specific BODIPY-based fluorescent probe for hypochlorite detection and cell imaging. Anal. Methods.

[27] Hong X., Wang Z., Yang J., Zheng Q., Zong S., Sheng Y., Zhu D., Tang C., Cui Y. (2012). Silylated BODIPY dyes and their use in dye-encapsulated silica nanoparticles with switchable emitting wavelengths for cellular imaging. Analyst.

[28] Zhan X., Sun N., Wu Z., Tu J., Yuan L., Tang X., Xie Y., Peng Q., Dong Y., Li Q. (2015). Polyphenylbenzene as a platform for deep-blue OLEDs: Aggregation enhanced emission and high external quantum efficiency of 3.98%. Chem. Mater..

[29] Tong H., Dong Y., Häußler M., Hong Y., Lam J.W.Y., Sung H.H.Y., Williams I.D., Kwok H.S., Tang B.Z. (2006). Molecular packing and aggregation-induced emission of 4-dicyanomethylene-2,6-distyryl-4*H*-pyran derivatives. Chem. Phys. Lett..

[30] Tracy H.J., Mullin J.L., Klooster W.T., Martin J.A., Haug J., Wallace S., Rudloe I., Watts K. (2005). Enhanced photoluminescence from group 14 metalloles in aggregated and solid solutions. Inorg. Chem..

[31] Ning Z., Chen Z., Zhang Q., Yan Y., Qian S., Cao Y., Tian H. (2007). Aggregation-induced emission (AIE)-active starburst triarylamine fluorophores as potential non-doped red emitters for organic light-emitting diodes and Cl_2_ gas chemodosimeter. Adv. Funct. Mater..

[32] Ow H., Larson D.R., Srivastava M., Baird B.A., Webb W.W., Wiesner U. (2005). Bright and stable core-shell fluorescent silica nanoparticles. Nano Lett..

